# Design of an Automated Mobile Phone-Based Reminder and Incentive System: Application in a Quasi-Randomized Controlled Trial to Improve the Timeliness of Childhood Vaccinations in Tanzania

**DOI:** 10.2196/65150

**Published:** 2025-09-10

**Authors:** Marco van Zwetselaar, Jan Ostermann, Melkiory Beti, Joy Noel Baumgartner, Sayoki Mfinanga, Esther Ngadaya, Lavanya Vasudevan, Nathan Thielman

**Affiliations:** 1 Zwets IT, Harskamp, The Netherlands; 2 Department of Health Services Policy and Management, Arnold School of Public Health, University of South Carolina, 915 Greene St, Columbia, SC, 29208, United States, 1 8037778747; 3 Kilimanjaro Clinical Research Institute, Moshi, United Republic of Tanzania; 4 School of Social Work, University North Carolina, Chapel Hill, NC, United States; 5 National Institute for Medical Research, Dar es Salaam, United Republic of Tanzania; 6 Hubert Department of Global Health, Rollins School of Public Health, Emory University, Atlanta, GA, United States; 7 Department of Medicine, Duke Global Health Institute, Duke University, Durham, NC, United States

**Keywords:** mHealth, automation, childhood vaccinations, SMS, Tanzania, open-source software, vaccination timeliness, digital health intervention, formative research, mobile phones

## Abstract

**Background:**

The global penetration of mobile phones has offered novel opportunities for communicating health-related information to individuals. A low-cost system that facilitates autonomous communication with individuals via mobile phones holds potential for expanding the reach of health messaging in settings with human resource and infrastructure limitations.

**Objective:**

We sought to design a flexible, low-code system using open-source software that could be adapted to different contexts and technical environments and accommodate a wide range of automation needs. We report on key details of the mobile phone–based appointment reminder and incentive system (mParis), document its use, review implementation challenges and adaptations to address these challenges in the context of a quasi-randomized trial of mobile phone–based reminders and incentives as means of increasing the timeliness of childhood vaccinations in Tanzania, and outline other use cases that highlight the versatility of the system.

**Methods:**

The mParis instance described in this paper, which is hosted in Tanzania, sent automated, individualized vaccination reminders in the form of SMS text messages to the mobile phones of mothers of young children. Process workflows, based on the national vaccination schedule of Tanzania, were programmed into mParis. Reminders for vaccinations due at ages 6, 10, and 14 weeks were sent 7 days and 1 day before and 14 days after each vaccination due date. A subset of messages included financial incentive offers to mothers for the timely vaccination of their children. We report on implementation outcomes, challenges, and adaptations to address these challenges.

**Results:**

Between August and December 2017, a total of 412 pregnant women were enrolled in the trial. After mothers reported the birth of their children, individualized vaccination reminder messages were sent for vaccination due dates between January and July 2018. From March 2018, messages contained financial incentive offers. Of 1397 messages sent, 1122 (80.3%) messages were recorded as delivered, 249 (18.8%) as expired and resent; 23 (1.6%) as failed, and 3 (0.2%) as sent but lacking a delivery confirmation. In total, 633 (45.3%) messages contained incentive offers. Of 173 women who received at least 1 message, 67 (38.7%) were sent reminders only; 106 (61.3%) women were sent at least 1 incentivized message. Numerous challenges were encountered during the system’s implementation, despite its deliberate design to accommodate basic problems, such as intermittent internet access and power failures. Continuous adaptation to increase the resilience of the system resulted in a successful deployment.

**Conclusions:**

mParis’ open-source nature, auditability, and ability to autonomously execute algorithms in a low-resource setting with frequent infrastructure challenges suggest favorable prospects to automate health communication in a wide range of settings. mParis’ use in other applications, including enrollment and follow-up for health-related research studies, demonstrates its versatility and ability to accommodate diverse challenges that may be encountered.

## Introduction

The global, nearly universal penetration of mobile phones has offered novel opportunities for engaging and retaining individuals in health-related interventions via electronic communication [1]. However, the large digital divide [2] between high- and low-resource settings has resulted in engagement and retention approaches that tend to either depend on robust and reliable technology infrastructure that does not exist in low-resource settings, or on costly human resources that are not scalable in high-resource settings [3]. Open-source systems that autonomously and flexibly execute algorithms even in the face of unreliable infrastructure, and that depend on human resources only to back-stop residual gaps, hold great potential for bridging the digital divide and are key to sustainability and scale-up [4,5].

The World Health Organization (WHO) global guidelines on digital interventions for health system strengthening recommend targeted client communication strategies using mobile phone communication modalities, such as text messaging [6]. In a systematic review [7] conducted to inform the guidelines, evidence was identified to support the use of targeted client communication in diverse health behaviors [8], including use of oral contraception, adherence to antiretroviral medication, and for supporting childhood vaccinations. In parallel, several challenges were identified related to equity in reach of mobile phone–based strategies, including gaps in mobile network coverage, lower mobile phone ownership among women, and gaps in literacy and digital health literacy. Combining targeted client communication strategies with digital tracking and clinical decision support may be beneficial for improving quality of care through better data documentation and use, although this may require greater resources to implement [9]. Thoughtful design with attention to scalability and equity is necessary to maximize the reach of mobile phone–based strategies to vulnerable populations, including women and those living in rural and remote areas.

Mobile phone–based strategies to support childhood vaccinations have seen a steady interest, with evidence for text message–based interventions dating back over a decade [10-13]. A systematic review by Mekonnen et al [14] found that SMS reminders significantly improved childhood vaccination coverage, demonstrating their effectiveness in diverse low- and middle-income country contexts. In Guatemala, Domek et al [15] conducted a randomized controlled trial showing that SMS reminders reduced delays in infant immunization visits and were well received by caregivers. Such digital strategies support the goals of the WHO Immunization Agenda 2030, which advocates for leveraging digital innovations to improve equitable vaccine coverage and strengthen health systems globally.

In previous research in Tanzania, we identified 3 primary challenges with the implementation of vaccination programs in rural settings: gaps in knowledge of vaccination recommendations, geographic barriers (lack of transportation options and costs of transportation) to accessing vaccination services, and gaps in cold chain infrastructure affecting vaccination service delivery [16]. In response to these challenges, we sought to examine the use of sending automated text messages that could address 2 of the 3 challenges: we sought to use personalized text messages to send reminders about upcoming vaccinations and deliver conditional incentives for timely vaccination to overcome geographic barriers [17].

The objectives of this study are to provide details on the formation, design, functionality, and technical features of the mobile phone–based appointment reminder and incentive system (mParis), describe the design and implementation of mParis to support timely childhood vaccinations in Tanzania, review implementation challenges and subsequent adaptations to address these challenges, and outline other use cases that highlight the versatility of the system to support research and intervention studies in both high- and low-resource settings.

## Methods

### Overview

We report here key details of mParis in accordance with guidelines for the reporting of health interventions using mobile phones: mERA (mHealth evidence reporting and assessment) checklist (Checklist 1) as well as the TiDieR (Template for Intervention Description and Replication) (Checklist 2) [18,19]. We illustrate the system’s versatility by describing its use in a quasi-randomized trial of mobile phone–based reminders and incentives as means of increasing the timeliness of routine childhood vaccinations in Tanzania [17]. The design of the system was guided by the study protocol and the national vaccination schedule in Tanzania. The process-driven system is particularly suitable to this application because it tracks the mother’s journey through the vaccination schedule of her child, which may include missed or delayed vaccinations that result in gaps in the vaccination schedule. We briefly discuss the broader applicability of the system to any scheduled events, especially when conditionality results in alternative process flows (eg, due to maternal or child death), and to other use cases, such as engaging and retaining participants in research studies. A parallel instance of mParis was used to send reminder messages to individuals participating in a research study focusing on HIV testing [20], and a third application of mParis currently delivers confidential, mobile phone–based HIV testing invitations to social network contacts of individuals seeking HIV testing or treatment [21].

### Technical Specifications of mParis

#### Software

**Figure 1.**
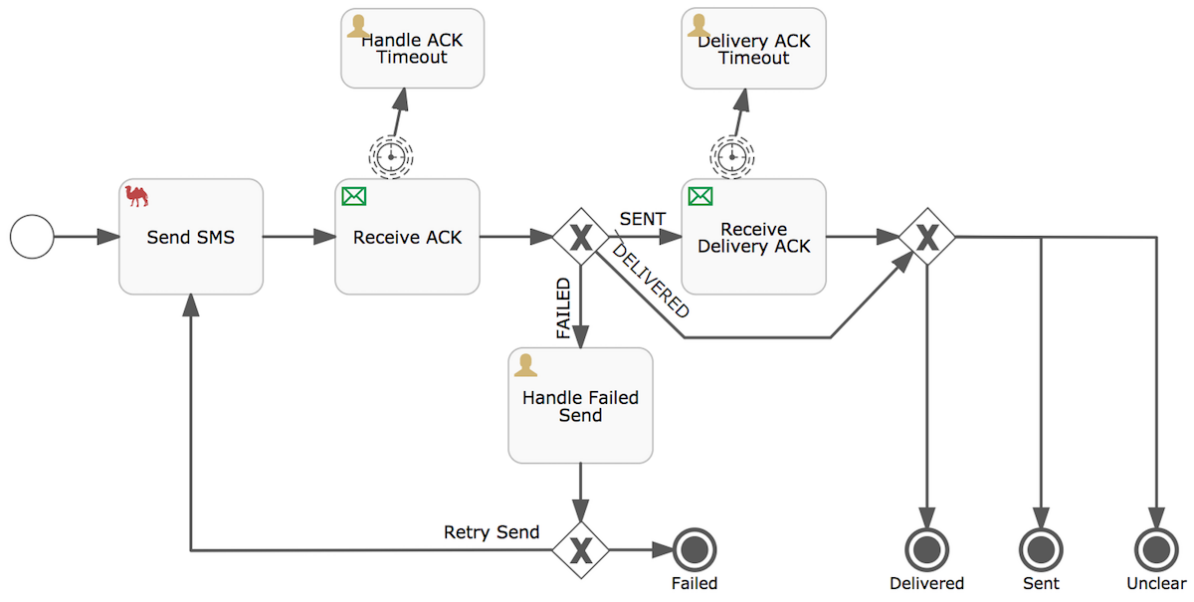
Process definition for the “Send SMS” process within mParis. This process diagram describes the sending of an SMS text message and results in either a “failed,” “delivered,” “sent,” or “unclear” status of the message. The process is started by a parent process. “x” indicates “or.”

mParis is a task scheduling, execution, and event tracking application built on Flowable [22], an open-source, Java-based workflow engine. The scheduling and execution of tasks are governed by intuitive, well-defined process definitions. Figure 1 illustrates this concept by showing a process definition for sending an SMS text message via mParis. The “Send SMS” process sends an SMS text message based on parameters that are specified by a parent process. In this example, the parent process prescribes the time at which the message should be sent, the recipient’s phone number, and the message text. After the SMS text message is sent, the process awaits an acknowledgement from the recipient’s SMS center, which can be either a confirmation that the message was sent, a delivery confirmation, or a send failure notification. Depending on the acknowledgment received, the process retries a failed message or continues to wait for a delivery confirmation. Finally, the process reports the status of the SMS text message (delivered, failed, sent, and unclear) to the parent process.

Figure 1 illustrates this concept by showing a process definition for sending an SMS text message via mParis. The “Send SMS” process sends a message based on parameters that are specified by a parent process. In this example, the parent process prescribes the time at which the SMS text message should be sent, the recipient’s phone number, and the message text. After the message is sent, the process awaits an acknowledgement from the recipient’s SMS center, which can be either a confirmation that the message was sent, a delivery confirmation, or a send failure notification. Depending on the acknowledgment received, the process retries a failed SMS text message or continues to wait for a delivery confirmation. Finally, the process reports the status of the SMS text message (delivered, failed, sent, and unclear) to the parent process.

Processes may trigger tasks, await task completion, or both. Tasks can be either robotic or human. Robotic tasks include, for example, the sending of SMS text messages or email messages, or the generation of reports. Human tasks include, for example, the handling of failure events, phone calls, or the completion of forms. Human tasks can be assigned to and claimed by users with prespecified roles and permissions. The system integrates events (eg, incoming messages) into the processes and tracks task completion.

Notably, Flowable is a relatively code-free (“low-code”) environment: process modeling can be done in an intuitive visual modeling environment using the standardized Business Process Modeling Notation (BPMN; version 2.0). Models execute directly in the Flowable framework. Depending on the application, front-end user screens (forms) may be added for user interactions. Processes are readily integrated with diverse back-ends, such as an SMS or email server. Data persistence is managed by the framework, which stores the values of process variables as they change throughout a process execution. Thus, there is no need to develop an underlying database.

#### Technology

Details of the technology comprising mParis are summarized in Table 1.

**Table 1.** Technology comprising the mParis instance used in a quasi-randomized trial of automated mobile phone–based vaccination reminders and incentive offers to mothers of newborn children (Tanzania, 2018).

Component	Details
Workflow engine	Flowable 6.3
Web proxy	Apache/2.4.7 and HTTPD server: https to public internet (Apache Software Foundation)
Application server	Apache Tomcat 8.5: mParis app, Flowable runtime framework and process modeling environment
Database	PostgreSQL 10 (PostgreSQL Global Development Group; process model and process instance storage)
Message broker	ActiveMQ 5.14.1 (Apache Software Foundation; guaranteed once-and-only-once event delivery between application server and SMS server)
SMS server	SMSTools3 using serial USB dongles for multiple providers
SIM card dongles	Airtel Tanzania SIM card
Redundancy	Redundant network providers, Uninterruptible power supply (UPS), offsite encrypted backup

#### Testing

Flowable facilitates testing of processes using unit tests and simulation, with support for accelerated timelines and mock events. mParis testing included, for example, the temporary removal of time-outs, and the use of keywords in mock SMS text messages to simulate specific events (eg, message failures, incoming messages, and missing delivery confirmations) or to trigger activities (eg, human tasks). A test server was set up to facilitate testing of the system from the perspective of all users. Reporting templates were developed to facilitate process evaluation in near real-time.

#### Security, Access, and Auditability

The instance of mParis described in this paper is hosted in the data center of the Kilimanjaro Clinical Research Institute (KCRI) in Moshi, Tanzania. Servers are hosted in server rooms with controlled physical access. Back-up to encrypted secondary (off-site) storage occurs daily. Another instance of mParis is hosted on a virtual server at Tanzania’s National Internet Data Center [23]. mParis can be accessed remotely over authenticated and encrypted network connections.

User roles are defined in the process modeler and assigned to user accounts. Roles determine the processes the user can initiate, and the tasks they can select from “team inboxes.” Users with administrator roles control account and role assignment. All events, including every change to process data, are fully auditable.

#### Cost

Flowable is open-source software freely available under the Apache License (version 2.0; Apache Software Foundation). Flowable’s low-code development allows for system adaptation and maintenance with limited programming. With the purchase of monthly SMS text message bundles, mParis can send large numbers of messages at minimal cost to any Tanzanian mobile phone number. Because SMS text message receipt is free in Tanzania, no cost is incurred by message recipients.

### Application: Reminders and Incentives for Timely Vaccinations

The first application of mParis was to support a quasi-randomized controlled trial in Mtwara Region in southern Tanzania; the study protocol and the characteristics of study participants, including their mobile phone use, have been previously described [17,24]. In this formative research study, we provide details on the implementation and adaptation of mParis in relation to the objectives of the trial, as well as the system’s functionality and extended use cases.

#### Study Context

In 2023, 21 million infants missed out on one or more life-saving vaccines [25], with the majority of “zero-dose” children living in the African region [26]. In Tanzania, only 53% of children aged 12‐23 months are estimated to be fully vaccinated against all basic antigens [27], and rates of vaccination timeliness are low [28,29]. Strategies that leverage digital health technologies (eg, vaccination appointment reminders) [14] have been shown to be effective at increasing vaccination coverage and timeliness. Our quasi-randomized trial aimed to evaluate the feasibility and potential efficacy of automated mobile phone–based vaccination reminders and conditional financial incentive offers for improving the timeliness of routine childhood vaccinations. The study was conducted in Mtwara Region in southern Tanzania, a region with large numbers of un- or undervaccinated children. We previously reported on high rates of mobile phone access and use in the study area [17]. At the time of the study, Tanzania reported 75 mobile phone subscriptions per 100 people; by 2022, the number had reached 92 subscriptions per 100 people [30].

#### Study Design

The trial compared three study arms: (1) standard of care (no reminders or incentives), (2) mobile phone–based reminders, and (3) mobile phone–based reminders with conditional financial incentive offers. The assignment to study arms was implemented at the level of the vaccination event. The primary study outcome was the timeliness of routine vaccinations for the newborn children, defined as vaccination receipt within 28 days of the scheduled vaccination due date, for vaccinations recommended at ages 6, 10, and 14 weeks.

#### Rationale

Reminders and incentives were evaluated as potentially scalable means of increasing the timeliness of routine childhood vaccinations. Reminders served as nudges [31], whereas conditional financial incentive offers served to offset transportation and other barriers that may prevent mothers from acting on their intention to get their children vaccinated on time [32].

#### Recruitment and Enrollment

To participate in the study, women had to be at least 16 years of age, in the third trimester of pregnancy, have access to a mobile phone, and provide informed consent. The target enrollment was 400 women from at least 4 urban and at least 8 rural health facilities and the surrounding communities. Health facilities within a 30 km radius of the regional capital that routinely provide childhood vaccinations were considered eligible venues for the recruitment of study participants.

A combination of purposive and snowball sampling strategies was used for recruitment. Eligible women presenting for antenatal care at participating facilities were approached by trained study personnel and offered enrollment in the study. Participating women and local community leaders were asked to identify other pregnant women in their community who, if eligible, were also offered enrollment in the study.

#### Use of mParis

**Figure 2.**
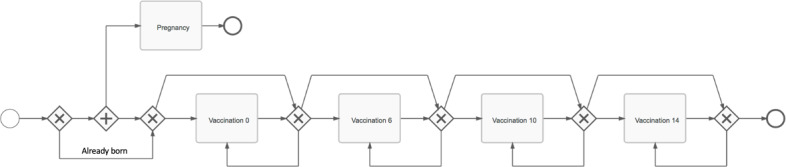
Process definition for the “Mother process” from study enrollment in pregnancy through the completion of the child’s 14-week vaccination. This process diagram describes an enrolled mother’s progression from pregnancy through vaccinations recommended at birth and ages 6, 10, and 14 weeks. The process is started by the enrollment process and invokes the pregnancy and vaccination sub-processes. “+” indicates “all,” while “x” indicates “or”. Participants were enrolled in Mtwara Region, Tanzania. The short message service-based intervention lasted from January to July 2018.

Pregnant women aged 16 years or older were enrolled into the study in their third trimester of pregnancy. Mobile phone numbers were documented for the women or anyone designated by the woman as a vaccine advocate, that is, someone who could relay information about upcoming vaccination appointments to the woman. The woman’s name, mobile phone number, and expected date of delivery were batch-uploaded into mParis, thereby triggering the mother process (Figure 2).

mParis sent reminders and incentive offers in the form of individualized SMS text messages (Table 2). Separate message templates were used for reminders before and after a vaccination due date, and for mothers and designees (ie, vaccine advocates). Reminders and incentive offers in the form of SMS text messages were found to be feasible and acceptable in this setting [24].

**Table 2.** Message templates for short message service messages sent via mParis during a quasi-randomized trial of automated mobile phone-based vaccination reminders and incentive offers to mothers of newborn children, by study arm and recipient type (Tanzania, 2018).

Arm, timing, and recipient		SMS message text^a^
Arm 2 (reminders)		
Before the due date		
Mother		Your child is due to receive a vaccination on [*due_date*].
Designee		Please remind [*mother_name*] that her child is due to be vaccinated on [*due_date*].
After the due date		
Mother		Has your child received the vaccination scheduled for [*due_date*]? If s/he has not been vaccinated, please bring her/him before [*end_date*].
Designee		Has [*mother_name*] brought her child to be vaccinated on [*due_date*]? If not, she should do so before [*end_date*].
Arm 3 (reminders and incentive offers)		
Before the due date		
Mother		Your child is due to receive a vaccination on [*due_date*]. If s/he receives the vaccination on time you will receive Sh2000.
Designee		Please remind [*mother_name*] that her child is due to be vaccinated on [*due_date*]. For a timely vaccination she will receive Sh2000.
After the due date		
Mother		Has your child received the vaccination scheduled for [*due_date*]? If s/he has not been vaccinated, please bring her/him before [*end_date*] to receive Sh1000.
Designee		Has [*mother_name*] brought her child to be vaccinated on [*due_date*]? If not, she should do so before [*end_date*] to receive Sh1000.

a Italicized content in square brackets represents a placeholder for message-specific information. All messages also contained the following information: “If s/he already received the vaccination call [*phone number*].” Message templates were translated to Kiswahili.

Recipients were study participants or their designees; messages were sent for routine childhood vaccinations with due dates between January and July 2018.

#### Process Definitions

After the report of a live birth, SMS reminder messages, in Kiswahili, were sent 1 week and 1 day before each vaccination due date (at 6, 10, and 14 weeks of age), and if no vaccination was reported, 14 days after the due date. For arm 3, reminders included a financial incentive offer for each timely vaccination.

A mother’s progression in the trial from enrollment during pregnancy through birth of the child, and the child’s vaccinations at birth and at ages 6, 10, and 14 weeks was described by the mother process (Figure 2). The mother process was started by the enrollment of a pregnant mother and invoked vaccination and other nested processes. Delays in the 6- and 10-week vaccination processes automatically shifted subsequent vaccination processes, that is, vaccination due dates for the 10- and 14-week vaccinations were set to 28 days after the previously completed vaccination.

**Figure 3.**
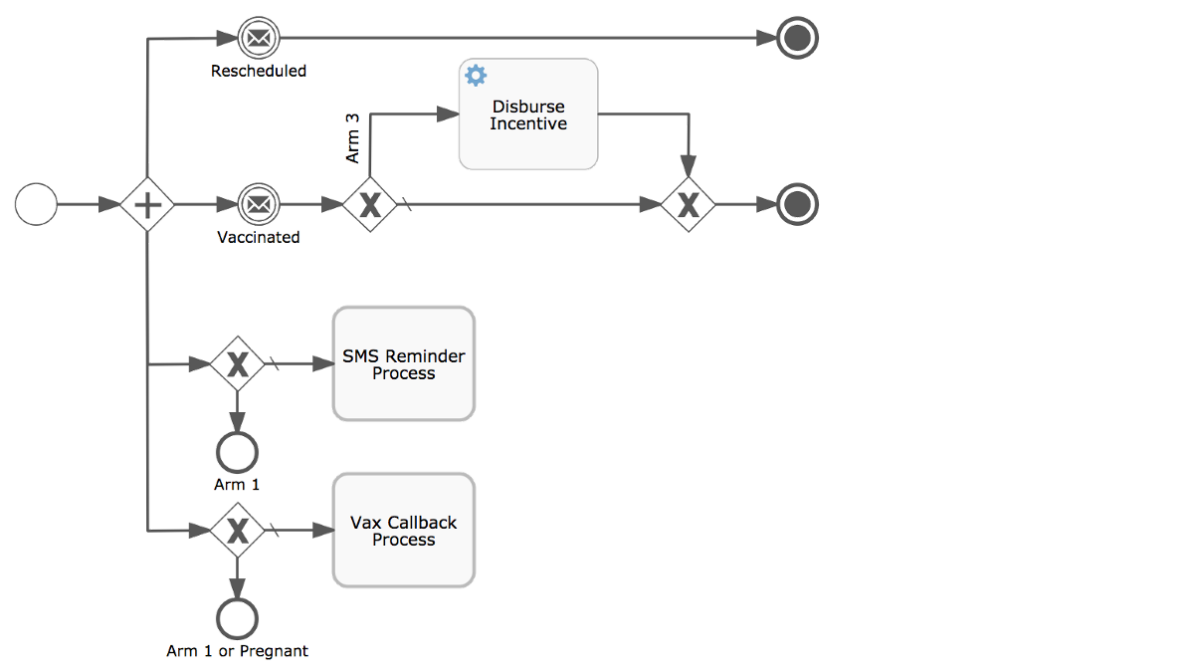
Process definition for the “Vaccination process” as a function of the study arm of the vaccination event. Notes: The vaccination process awaits notification of a received or rescheduled vaccination and delegates the study-arm specific sending of SMS messages, phone calls, and incentive payouts to child processes. “+” indicates “all,” while “x” indicates “or.”

The “vaccination process” (Figure 3) illustrates the versatility of the workflow approach, and thus the mParis system. Study-arm specific vaccination events required a process definition that flexibly supported study arm–specific invocations of reminder, call-back (phone call), and incentive disbursement processes. The result of the vaccination process is reported back to the mother process and results either in the restart of the vaccination process (in the case of a rescheduled vaccination), progression to the next vaccination (in the case of a completed vaccination), or the end of the mother process (in the case of a completed 14-week vaccination).

#### Implementation Details

The content of the messages was pilot tested with 32 women, and iterative changes were made to optimize comprehension, content, and clarity. Message templates (Table 2) were stored in a properties file, with placeholders that allowed for the individualization of message content to each recipient (eg, mother’s name and vaccination due date).

Children’s birth dates, dates of completed vaccinations, and scheduled and rescheduled vaccination due dates were ascertained by research assistants who regularly contacted mothers via mobile phone–based follow-up. These dates were used to iteratively update active mParis processes until process completion, which occurred upon completion of the 14-week vaccination or the end of the follow-up interview, whichever came first.

#### Ethical Considerations

The protocol was approved by the institutional review boards at Duke University (protocol 2017‐0591) and the University of South Carolina (facilitated review, Pro00051213), United States, and the National Institute for Medical Research (NIMR) in Tanzania (NIMR/HQ/R.8a/Vol. IX/2194). The trial was registered with ClinicalTrials.gov (NCT03252288). Written informed consent was obtained from all study participants. Participants received a compensation of TSH 3000 after the completion of each survey. At the time of the study, US $1 was worth approximately TSH 2220. Password-protected tablet devices were used to collect survey data; analytic data are stored on encrypted, password-protected devices. Identifying information was collected, transferred, and stored separately from survey data. The security of the mParis system is described above.

## Results

### Implementation

Between August 15, 2017, and December 8, 2017, a total of 412 pregnant women in their last trimester of pregnancy were enrolled into the study. Neither reminders nor incentive offers were sent for vaccination due dates through January 11, 2018 (arm 1; “control” phase). During the control phase, 140 women (34% of the sample) were confirmed to have completed the vaccination process; 14 (3.4%) were withdrawn due to maternal or child death; 79 (19.2%) women were lost to follow-up.

Vaccination reminders were automatically sent for vaccination due dates between January 12, 2018, to July 10, 2018. In total, during the 6-month implementation period, 1397 SMS text messages were scheduled to be sent through mParis. Of these, 1122 (80.3%) messages were recorded as delivered, 249 (18.8%) as expired (these were automatically resent), 23 (1.6%) as failed, and 3 (0.2%) as sent but lacking a delivery confirmation.

Women with known vaccination due dates between January 12, 2018, and March 5, 2018, were sent reminder messages (arm 2). Women with known vaccination due dates between March 6, 2018, and July 10, 2018, received reminder messages with tiered incentive offers for timely vaccinations (arm 3). Of 173 women who received at least 1 message, 67 (38.7%) women were sent reminders only, whereas 106 (61.3%) women were sent at least 1 incentivized message. In total, 633 (45.3%) messages contained a financial incentive offer for the timely vaccination of the child.

### Challenges, Adaptations, and Resolutions

Multiple challenges were encountered during the implementation of mParis, despite the system’s deliberate design to a priori accommodate basic problems, such as intermittent internet access and power failures. Key challenges are summarized in Table 3, with illustrative sample quotes from communications between the study team and the system developer. Formative, continuous adaptation of the system to increase the resilience of the system, complemented by limited manualized backstops during trial implementation, enabled the successful deployment of a robust system capable of accommodating diverse challenges that could be encountered in varied settings.

**Table 3.** Challenges encountered in the implementation of mParis in the context of the quasi-randomized trial (Tanzania, 2018).

Challenge	Description and sample quotes from communication between the study team and the system developer	Resolutions
Unreliable electricity	Heavy rains cause disruptions in electricity supply and internet connectivity. “When there are longer power cuts in Moshi, [network provider’s] network machinery goes down and we lose the connection. Very frustrating, as it’s outside of our control, and very frequent now with the heavy rains*.*”	Server is plugged into an uninterruptible power supply (UPS) unit, UPS is backed by a generator. SMS text messages are received and sent from a local server, with no dependence on internet connectivity.
Accessing mParis via the internet	Disruptions in the server’s internet connectivity led to inaccessibility of mParis via the internet. “The issue with mPARIS is the connection between us and the internet exchange downtown.” “Or... again ... someone forgot to pay the bill.” “… when I tried to switch over to our dedicated DSL line it turned out that [internet provider] did not assign the promised static IP but gave us a dynamic IP instead.”	Redundant internet connections: “I’m thinking about alternatives (wireless broadband over VPN to an [external vendor] endpoint)” Batch export of human tasks: “I have a fairly good redundancy set up now -- each day I pull the status reports from mParis, process them, and send a spreadsheet with priority callbacks … so they can work online as well as offline.”
Flowable versioning	“I decided to go with the latest Flowable release (6.3.0 just out), but they’ve overhauled the way they do configuration.”	Additional testing after version changes.
Lost SMS text messages	Unannounced change in provider-imposed daily limit on the number of SMS text messages that could be sent from a single SIM card.	Allocate nontime-sensitive messages to different days; distribute load over multiple modems. Shorten message templates to stay within the SMS text message character limit. Alternatively, contract with multiple SMS providers.
Support for multiple mobile phone carriers	Not all modems support Linux. “I got a [network provider] SIM this weekend, with (sigh) a cheap imitation dongle because they ran out of the proper ones. Sure enough, despite what it says on the box it doesn’t work on Linux or MacOS…”	Testing of diverse modems, including “legacy dongles”. “Luckily I found my sturdy old [mobile phone provider] dongle (dated 2009) and it works fine ... I’ll look for two more oldies.”
A large number of processes to be launched	The large number of study participants and unreliable internet connectivity made the individualized registration of participants in mParis and the individualized launch of the respective “mother processes” inefficient.	Added functionality for batch uploads. “For the initial entry, I think it’ll be easy for me to convert a CSV table to REST calls and shoot those at the system from here (Flowable exposes pretty much all functionality it has also over REST).”
Time sensitivity of SMS reminders	Late reports of prior vaccinations and unsent or failed reminder messages required the dynamic and highly time-sensitive rescheduling of SMS reminder messages in accordance with the study protocol.	Added functionality allowed for the instantaneous sending of relevant SMS reminder messages for individual study participants.

a SMS-short message service. Source: email record between the system developer and the study team.

### Other mParis Use Cases

Since 2018, 2 other instances of mParis have supported research studies in Tanzania, and a third use case is currently being developed. Between 2018 and 2020, mParis was used to “batch-send” recruitment reminders to support enrollment into a randomized controlled trial aimed at improving uptake of HIV testing in northern Tanzania [3]. During the recruitment of potential participants at off-site recruitment venues, eligible individuals provided contact phone numbers to members of the research team. mParis sent follow-up messages to these individuals, inviting them to come to the study’s research offices for consent and enrollment. Subsequently, for participants enrolled in the study, mParis sent scheduled SMS reminders to return to the research offices for follow-up. mParis also sent reminder messages to participants who were given HIV testing invitation cards to get tested for HIV as described on the cards.

mParis is currently supporting a randomized trial to evaluate the acceptability, efficacy, and cost-effectiveness of confidential SMS-based social network referrals for HIV testing as a means of reaching high-risk individuals and prompting them to test for HIV [21]. Clients presenting for HIV testing or treatment are offered the opportunity to send confidential SMS-based HIV testing invitations to social network contacts (eg, friends, family members, and coworkers) who could benefit from HIV testing. Inviters can choose from a menu of message options; mParis autonomously schedules invitation messages to be sent to invitees’ mobile phones after 1 and 7 days. The system ensures that an inviter cannot be identified by their invitee, that no invitee phone number receives more than 2 messages regardless of how often they are invited, and that failed messages are automatically resent.

mParis is presently being adapted to support a cluster-randomized trial of an intervention aimed at improving the timeliness of routine childhood vaccinations in rural western Tanzania. mParis will be integrated with Dimagi’s CommCare system [33] to support community health care workers to remind mothers of upcoming vaccination due dates, notify mothers of vaccine stock-outs, and mitigate vaccination-related knowledge gaps. These applications exemplify the versatility of the system, which can be adapted to support other health interventions (eg, messages to support medication adherence), extended beyond SMS technology (eg, to WhatsApp, Meta, or email communication), and even to support mobile phone–based survey research (eg, via Unstructured Supplementary Service Data [USSD]).

## Discussion

### Principal Findings

In this study, we describe the design of a highly versatile mobile phone–based appointment reminder and incentive system (“mParis”) to facilitate autonomous communication with individuals. We provide a detailed description of a specific instance implemented in a low-resource setting to support the timely vaccination of children. mParis autonomously sent more than 1300 individualized SMS text messages to Tanzanian mothers participating in a research study aimed at improving the timeliness of routine childhood vaccinations. We detail the technical architecture, demonstrate mParis’ technical feasibility, assess implementation challenges, and explore its broader applicability to public health and research contexts.

mParis, built on a business process modeling platform, combines several features [34] that increase its potential applicability to other contexts. First, the system is built in Flowable, an open-source, low-code modeling environment. Intuitive and highly flexible process definitions allow for the specification of diverse algorithms with minimal programming requirements. The low-code nature of the system allows for workflow logic to be visually specified, with no “hand coding” needed, and to be executed and supervised by the platform. This ensures that the system can be adapted with limited programming expertise and that processes are executed in full alignment with their design. Second, the system maintains consistency, that is, every process instance is in a well-defined state at each moment (comparable with the way databases guarantee data consistency) and is fully auditable (ie, it keeps track of every event and action that occurs in a process). These features support operational efficiency and minimize the need for coordination between stakeholders. For example, if multiple team members jointly process events (such as call-backs to mothers), there is instantaneous tracking of events, minimizing the need for additional event-related communication. Finally, the system is open source, with many integration components available to connect to external services. For example, while mParis’ primary task in the sample application comprised the algorithmic sending of SMS text messages, the system may be integrated with other popular communication platforms, such as WhatsApp and Facebook Messenger (Meta), or WeChat (Tencent). Similarly, mParis is readily integrable with existing health information systems; its robust security infrastructure and full auditability suggest that an appropriate integration can be fully compliant with HIPAA (Health Insurance Portability and Accountability Act) rules and regulations. The mParis instance and application described in this paper was subject to several limitations. Some of these limitations were addressed, as described. Others are being addressed in ongoing use cases or may be addressed in future use cases.

Most notably, the mParis application described here relied on a dongle to send SMS text messages. A larger-scale implementation of the system requires additional capacity for sending messages and would benefit from a dedicated short message peer-to-peer connection, though there are no fundamental limits to the scale of the existing system.

Second, reminders and incentive offers were sent using SMS text messages. SMS technology is not secure and, due to restrictions in message type and length, may not be optimal for many use cases. mParis allows for the integration of other modes of communication, such as emails or WhatsApp messages. These were not considered feasible in the application described here. Mobile-cellular subscription rates are high in Tanzania at 92 per 100 people [30]; however, rural populations have lower smartphone access than urban populations, and a majority with phones rely on basic communication strategies, such as phone calls and SMS text messages [24,35].

Third, while at enrollment into the trial, more than 98% of women enrolled in this study had used a mobile phone to make phone calls, only two thirds had used mobile phones to send or receive SMS text messages, and two thirds of women reported using a mobile phone less than once a week [24]. Thus, despite its reliance on basic SMS-based communication, mParis may not have been able to overcome all access barriers and may need manualized back-stops, such as phone calls, to reach all intended recipients within a timely manner. However, by fully automating reminders and incentive offers for those with mobile phone access, limited human resources can be targeted toward back-stopping residual gaps. This highlights the broad usage potential of this highly versatile system for autonomously engaging individuals across a variety of contexts.

In summary, mParis was successfully deployed and used in a quasi-randomized trial of SMS reminders and incentives as means of increasing the timeliness of childhood vaccinations in Tanzania. Iterative adaptations to mParis in response to stochastic environmental and technical implementation barriers resulted in the formation of a robust mHealth system that is locally hosted in a low-resource setting, but readily extensible to other geographic and topical contexts, including high-resource settings.

### Conclusion

mParis’ open-source nature, auditability, and ability to autonomously execute algorithms in the face of diverse challenges suggest favorable prospects for the automation of critical health communication and research-related tasks in a wide range of settings. These characteristics are key to the sustainability and scale-up of mHealth systems in both high- and low-resource settings.

## Supplementary material

10.2196/65150 Checklist 1 mERA (mHealth Evidence Reporting and Assessment) checklist.

10.2196/65150 Checklist 2 TiDieR (Template for Intervention Description and Replication) checklist.

## Abbreviations

BPMN: Business Process Modeling Notation
HIPAA: Health Insurance Portability and Accountability Act
KCRI: Kilimanjaro Clinical Research Institute
mERA: mHealth evidence reporting and assessment
mParis: mobile phone-based appointment reminder and incentive system
NIMR: National Institute for Medical Research
TiDieR: Template for Intervention Description and Replication
USSD: Unstructured Supplementary Service Data
WHO: World Health Organization
